# The Characteristics of Thrombin in Osteoarthritic Pathogenesis and Treatment

**DOI:** 10.1155/2014/407518

**Published:** 2014-09-16

**Authors:** Pei-Yu Chou, Chen-Ming Su, Chun-Yin Huang, Chih-Hsin Tang

**Affiliations:** ^1^Graduate Institute of Basic Medical Science, China Medical University, No. 91, Hsueh-Shih Road, Taichung, Taiwan; ^2^Department of Nursing, Hung Kuang University, Taichung, Taiwan; ^3^Department of Orthopaedic Surgery, China Medical University Beigang Hospital, Yun-Lin County, Taiwan; ^4^Graduate Institute of Clinical Medical Science, China Medical University, Taichung, Taiwan; ^5^Department of Pharmacology, School of Medicine, China Medical University, Taichung, Taiwan; ^6^Department of Biotechnology, College of Health Science, Asia University, Taichung, Taiwan

## Abstract

Osteoarthritis (OA) is a mechanical abnormality associated with degradation of joints. It is characterized by chronic, progressive degeneration of articular cartilage, abnormalities of bone, and synovial change. The most common symptom of OA is local inflammation resulting from exogenous stress or endogenous abnormal cytokines. Additionally, OA is associated with local and/or systemic activation of coagulation and anticoagulation pathways. Thrombin plays an important role in the stimulation of fibrin deposition and the proinflammatory processes in OA. Thrombin mediates hemostatic and inflammatory responses and guides the immune response to tissue damage. Thrombin activates intracellular signaling pathways by interacting with transmembrane domain G protein coupled receptors (GPCRs), known as protease-activated receptors (PARs). In pathogenic mechanisms, PARs have been implicated in the development of acute and chronic inflammatory responses in OA. Therefore, discovery of thrombin signaling pathways would help us to understand the mechanism of OA pathogenesis and lead us to develop therapeutic drugs in the future.

## 1. Introduction

Osteoarthritis (OA) is a mechanical abnormality associated with degradation of joints, including articular cartilage, synovial fluid, and subchondral bone [1, 2]. As per the American College of Rheumatology, 70% of people over the age of 70 years have X-ray evidence of OA [3]. Patients with OA initially begin with adequate fluid content and healthy cartilage but gradually deteriorate over time, showing progressively decreased joint composition. Therefore, OA is termed as a degenerative joint disease [4]. Since several factors can contribute to development of OA, such as overuse of joints or exogenous stress, anyone can develop the disease [5].

## 2. Osteoarthritis

OA is characterized as a chronic, progressive degeneration of articular cartilage, abnormalities of bone, and synovial changes. Typical characteristics of OA include progressive loss of articular cartilage resulting in structural and functional failure of joints. Since cartilage plays an important role as a cushion within the joint, loss of articular cartilage causes intolerable pain. The most common symptom of OA is local inflammation resulting from exogenous stress or endogenous, abnormal cytokines [6, 7]. Normal cartilage matrix is mainly composed of type II collagen, which provides tensile support for the tissue [8], in addition to proteoglycans, and chondrocytes [9]. It has been reported that matrix metalloproteinase (MMP) is overexpressed in patients with OA resulting in cleavage of collagen and proteoglycans from the matrix [10]. In addition, numerous studies have verified abnormal synovium within the OA joint as compared to normal synovium, which is shown adequate blood and nerve supply. These abnormalities include thickening of the lining layer, increased vascularity, inflammatory cell infiltration leading to local synovitis, hypertrophy, and thickening of the joint capsule [11]. Treatment options for OA focus on pain relief and reducing inflammation. Therefore, traditional treatment for OA includes nonsteroidal anti-inflammatory drugs (NSAID), analgesics, and steroid injections that are used to treat pain and inflammation [12, 13]. Since the exact etiology of OA is not well understood, biochemical markers could help us better understand the pathogenesis of OA and design new therapeutic approaches for the treatment of the OA.

## 3. Characteristics of Thrombin

Thrombin, also known as blood-coagulation factor IIa (FIIa), and its inactive precursor prothrombin, also called coagulation factor II (FII), are serine proteases and members of the family of vitamin K-dependent coagulation factors. The zymogen prothrombin is enzymatically cleaved by the prothrombinase complex through the activated platelet phospholipids factor Xa (FXa) and factor Va (FVa) [14, 15]. Thrombin is essential for homeostasis, thrombosis, and inflammation triggered by tissue damage. Thrombin has two important functions: coagulation and anticoagulation. Additionally, arthritis is involved in local and/or systemic activation of coagulation and anticoagulation pathways. However, during coagulation, thrombin present in the blood can result in widespread thrombosis and cause a reduction in blood flow [16]. Bokarewa and colleagues demonstrated that tissue-factor (TF) is expressed in endothelial cells producing tumor necrosis factor (TNF) and interleukin-1 (IL-1) and in monocytes inducing chemokines, such as macrophage inflammatory protein 1 (MIP-1) and chemokine (C-C motif) ligand 5 (CCL5) [17, 18]. Thus, TF is considered to appear as a result of inflammation and triggers the immune response and coagulation systems. Thrombin is not only a mitogen but also a potent vasoconstrictor, causing local vasoconstriction, which directly stimulates vascular smooth muscle and adrenergic receptors. Thrombin is involved in tissue repair, activation of platelet and endothelial cells, and inflammation by stimulating deposition of fibrin. As part of its coagulation function, thrombin stimulates fibrin deposition, thus influencing inflammation, and activates transglutaminase factor XIIIa to convert soluble fibrinogen into an insoluble fibrin clot [19]. Fibrin is a ligand for intercellular adhesion molecule-1 (ICAM-1, CD54), CD11b/CD18 (CR3, Mac-1), and CD11c/CD18 (CR4, p150/95). Fibrin binds to α/*β* integrins to promote adhesion and migration of leucocytes, followed by accumulation of leukocytes in the matrix during inflammation [20–22]. Direct injection of thrombin has been shown to stimulate peritoneal accumulation of IL-6 and MCP-1 in a fibrin-dependent manner [23]. Thus, thrombin regulates fibrin to induce chemokines/cytokines during inflammation.

Thrombin participates in anticoagulation, also called fibrinolysis, during the process of inflammation by forming a complex with thrombomodulin (TM), an integral membrane protein expressed in endothelial cells. The thrombin-TM complex activates protein C (PC), an inhibitor of the coagulation cascade, which binds to protein S, and, in turn, this stops the process of coagulation. Besides, this complex also activates thrombin activatable fibrinolysis inhibitor (TAFI) to prolong clot lysis and inhibit coagulation via removing terminal lysine residues from fibrin [24, 25]. For TF-induced coagulation, thrombin also activates TF pathway inhibitor (TFPI) by inhibiting prothrombinase complexes from initiating coagulation [26–29]. Hence, thrombin-TM complex also inhibits fibrinolysis by the profibrinolytic effect of anticoagulation proteases activated protein C (APC), which causes further anticoagulation. Overall, thrombin does not simply act as a mediator of coagulation or anticoagulation, but rather as a signaling molecule to control inflammation.

## 4. Thrombin-Dependent Signaling and Protease-Activated Receptors

Thrombin promotes platelet activation and aggregation via activation of protease-activated receptors (PARs) on the platelet surface. Thrombin activates intracellular signaling pathways by interacting with transmembrane domain G protein coupled receptors (GPCRs), also known as PARs. There are four members of the PAR family, namely, PAR-1, PAR-2, PAR-3, and PAR-4. Thrombin activates PAR-1, PAR-3, and PAR-4, but not PAR-2 [16, 30]. Thrombin or serine proteinases activate also PARs by cleaving an N-terminal peptide bond and generating a new N-terminus, which acts as a ligand for transmembrane receptors to induce signal transduction [31–33]. Thus, PARs undergo conformational changes to couple with heterotrimeric G proteins, of which, PAR-1 couples with G protein subtypes, such as Gα_*q*_, Gα_12/13_, and Gα_*i*_ to activate mitogen-activated protein kinase (MAPK) cascades (Table 1) [31, 34, 35]. PAR-1 is also activated by coagulation FXa as well as APC, matrix metalloproteinase-1 (MMP-1), neutrophil elastase (NE), and neutrophil proteinase-3 (PR3) [31]. PAR-3 and PAR-4 are mainly activated by thrombin, but PAR-4 can also be activated by cathepsin G, a protease secreted by neutrophils [35, 36]. Although PAR-3 acts as a cofactor binding to thrombin to activate PAR-4 in rat, its mechanism of action in humans remains unclear [31, 35, 37]. PAR-2 is insensitive to thrombin; however, it can be activated by serine proteinases, mast cell tryptase, and allergic or bacterial proteases. Arrestin, a PAR2-selective agonist, is suggested to support extracellular regulated kinases 1/2 (ERK1/2) signaling in the cytoplasm, independently of G-protein activation [35, 38].

PARs are not only highly expressed on platelets, but also found on endothelial cells, monocytes, fibroblasts, T-lymphocytes, smooth muscle cells, and certain tumor cells [16, 31]. Activation of PARs on platelets is critical for hemostasis and thrombosis. For endothelial cells, thrombin-induced endothelial cell hyperpermeability results in recruitment of immune cells and release of growth factors and cytokines [39, 40]. Thrombin also induces release of calcium through RhoA/Rho kinase pathway and activation of myosin light chain (MLC) kinase to inhibit myosin phosphatase, causing a disruption of endothelial barrier function through interactions with actin-myosin [16, 34]. Thrombin-induced protein-tyrosine kinase (PTK) affects the phosphorylation of junctional proteins, vascular endothelial-cadherin (VE-cadherin), and catenins and results in migration and proliferation in several tumors such as lung, colon, and gastric cancer [35, 41–43]. Previous studies have implicated PARs in the development of acute and chronic inflammatory responses as well as thrombin [16, 31, 35]. The role of PAR-1 in inflammation has been clearly established, in vasodilation and mast cell degranulation, which increase the production of cytokines or chemokines via PAR-1, and in the adhesion of macrophage via fibrin [23]. Thrombin-regulated chemokine production also triggers calcium release in monocytes [44]. Stimulation of isolated monocytes by thrombin or a receptor-selective PAR1-AP (TFLLRNPNDK) causes upregulation of monocyte chemotactic protein-1 (MCP-1) [44] or release of IL-6, respectively [45, 46]. Taken together, these results suggest that thrombin mediates hemostatic and inflammatory responses and guides the immune response to tissue damage. Therefore, thrombin receptors involved in regulation of signaling cascades may provide a therapeutic target for inflammation.

## 5. Thrombin as a Signaling Factor Regulating OA Progression

Although OA is not considered systemic inflammatory disease, joint inflammation resulting from a local increase in synovial membrane vascularity or cartilage degradation is commonly associated with OA. OA is usually classified as a noninflammatory disorder. However, Thrombin is known to be involved in the regulation of fibrin deposition, cell migration, cell invasion, and proinflammatory processes. A number of growth factors and chemokines/cytokines have been found to regulate the expression of thrombin in many pathological states, indicating a critical role in OA progression. Therefore, we discuss different key events during OA pathogenesis, including cartilage degradation, synovial membrane abnormalities, chemokine/cytokine cascades, and production of inflammatory mediators such as heme oxygenase (HO).

### 5.1. Chemokines/Cytokines

Numerous chemokines/cytokines have been found to regulate expression of thrombin in OA [23]. Chemokine (C-C motif) ligand-2 (CCL2)/MCP-1 are known to be expressed in OA and rheumatoid arthritis [47, 48]. CCL2 recruits monocytes, memory T cells, and dendritic cells to the site of inflammation that is triggered by tissue damage [49]. Recently, expression of CCL2 has been detected in pathological arthritis via macrophage aggregation [50]. A study further suggested that thrombin induced CCL2 production via the PAR-1 mediated c-Src/MEK/ERK-dependent signaling pathway in human osteoblasts (Table 1) [48]. Thrombin also increased the expression of inflammatory cytokines such as IL-1*β*, TNF-α, fibroblast growth factor-1/-2 (FGF-1/-2), transforming growth factor-*β*1 (TGF-*β*1), IL-6, connective tissue growth factor (CTGF), tenascin C (TnC), and cyclooxygenase-2 (COX-2) in osteoblasts or osteoblast-like cells (Table 1) [51–54]. Thus, thrombin was shown to induce the release of proinflammatory cytokines via PAR-1 on the surface of osteoblasts. However, Pagel and colleagues found that earlier stages of bone repair are delayed in mice lacking PAR-1 [55]. Taken together, these results suggest that thrombin-induced activation of various chemokines/cytokines via PAR-1 may be one key mechanism of inflammation in OA.

### 5.2. Cartilage

It is necessary to conserve structural and functional integrity of the bone tissue during normal physiological conditions. Chondrocytes can maintain equilibrium between anabolic and catabolic activities in cartilage [10]. It is not yet completely understood which factors are responsible for initiating the degradation and loss of articular tissue. Thrombin is a key mediator to release proteoglycan in the degradation of human and bovine cartilage [56]. In OA, chondrocytes increase production of various proteolytic enzymes such as aggrecanases and MMP, resulting in aberrant cartilage destruction [10]. It has been shown previously that MMP-13 is overexpressed in OA [57, 58], which caused degradation of articular cartilage components, such as types II, IV, and IX collagen and proteoglycan [59]. Shiomi et al. showed that specific deletion of MMP-13 (gene) or inhibition of MMP-13 activity in cartilage decelerated aggressive OA (Table 1) [59]. Therefore, Huang et al. investigated the intracellular signaling pathways involved in thrombin-induced MMP-13 expression using human chondrocytes, which showed reduced MMP-13 production after transfection with siRNAs against PAR1 and PAR3, but not PAR4. The result also found that PAR1/PAR3 receptors activation by MMP-13 is increased by thrombin via a mechanism involving EGFR transactivation and activation of PKC*δ*, c-Src, PI3K, Akt, and finally AP-1 on the MMP-13 promoter, thereby contributing to cartilage destruction during arthritis [60]. HO is another inflammatory mediator consisting of three isoforms: HO-1, HO-2, and a less-characterized HO-3 [61]. HO-1 is inducible in response to stress such as reactive oxygen species, nitric oxide, heat shock, and hypoxia, and it also protects against oxidative injury and decreases inflammation [62, 63]. It has reported that HO-1 could inhibit cartilage erosion accompanied by extensive fibrosis in the joint [64]. In animal arthritis models, the levels of TNF-α, IL-2, and IL-10 were decreased by the HO-1 inducer cobalt protoporphyrin IX (CoPP), indicating that HO-1 deficiency causes chronic inflammatory conditions in arthritis. Moreover, HO-1 induced by the clinical vasodilator, sodium nitroprusside, inhibits apoptosis of arthritic chondrocytes through ERK inhibition and p38 activation to decrease activity of MMP-1 and MMP-13 (Table 1) [65]. These findings show that thrombin-activated cartilage destruction in OA pathogenesis occurs via MMP-13.

### 5.3. Synovium

Once OA onset occurs, macrophages can invade or migrate into a joint. The macrophages then release large amounts of proinflammatory and procatabolic mediators into the synovium [66]. In addition, thrombin-derived from the synovial fluids of OA patients also mediated production of proinflammatory factors and was characterized as a marker of synovitis [67]. Synovitis is secondary to cartilage degradation, which occurs in the adjacent, damaged cartilage. This inflammation is characterized by large amounts of proinflammatory and procatabolic mediators and a local increase in synovial membrane vascularity. In advanced OA, synovitis extends into the synovial membrane and progresses to fibrosis and villi hypertrophy [66]. In synovial cells, HO-1 is an important regulator of inflammation and affects cartilage degradation. Synovial membrane from OA patients has been found to produce IL-1*β* and TNF-α. Presence of chronic inflammatory factors and proinflammatory cytokines are a feature of synovial membranes from patients with early OA [68, 69]. However, the exact mechanism of macrophage-derived proinflammatory cytokine production in arthritic synovium is not well understood. Previous studies found that OA synovial fibroblasts (OASFs) showed significantly higher expression of thrombin than normal synovial fibroblasts. Furthermore, it was shown that thrombin induced concentration- and time-dependent expression of HO-1 in OASFs via PAR-1, PAR-3, and PKC*δ*/c-Src signaling pathways [70, 71]. In pharmacologic inhibitors, thrombin-regulated HO-1 expression was attenuated by thrombin inhibitor, d-phenylalanyl-l-prolyl-l-arginine chloromethyl ketone (PPACK), PKC*δ* inhibitor (rottlerin), or c-Src inhibitor (PP2), suggesting a hint that thrombin is involved in upregulation of HO-1. Besides, expression of nuclear factor erythroid-2-related factor (Nrf2) also contributes to thrombin-induced HO-1 production in OASFs (Table 1) [72]. Consequently, the discovery of thrombin-mediated HO-1 expression clarified the mechanism of OA pathogenesis and may lead to the development of more effective therapeutic targets for OA treatment in the future.

## 6. Thrombin as a Therapeutic Target in OA

Given the important role of thrombin-mediated signaling in OA progression, there has been increasing interest in therapeutic strategies to target this protein. In the cartilage or synovium, increased expression of thrombin and downstream intracellular signaling pathway via transmembrane domain GPCRs, PARs, are closely associated with inflammation and its mediators. Signaling via PARs may also promote OA progression by inducing inflammation in osteoblasts, chondrocytes, and OASFs. Moreover, some studies indicate that thrombin acts as a mitogen to stimulate the abnormal proliferation of synovial cells during OA pathogenesis [73, 74]. The aim of this review is to summarize the mechanism of thrombin-mediated OA pathogenesis and develop therapeutic approaches for treatment of OA. These include thrombin antagonists and herbal medicines.

### 6.1. Thrombin Antagonists

Thrombin promotes fibrin formation and protein C activation. Factor XIII is activated by thrombin that stabilizes fibrin complex and stimulates platelet, which helps in clotting. On the other hand, thrombin interacts with thrombomodulin and activates protein C and TAFI to initiate anticoagulation and inhibit fibrinogenesis. Given its broad-spectrum activities, thrombin represents a good target for anticoagulant drugs such as heparin, warfarin, and direct thrombin inhibitors (DTIs) [75, 76]. Thrombin has three binding sites: the active site, exosite 1, and exosite 2. The exosite 1 is anion-binding and binds to fibrin and exosite 2 is a heparin-binding domain [75, 77]. Some drugs bind to either exosite 1 or exosite 2 and may influence activity at the active site. DTIs are anticoagulant drugs that are commonly used to prevent the blood clot formation by directly inhibiting thrombin. DTIs inhibit thrombin via two mechanisms: (1) where bivalent DTIs simultaneously block the active site and exosite 1, thus acting as competitive inhibitors of fibrin, and (2) where univalent DTIs block only the active site; both types of DTIs inhibit unbound and fibrin-bound thrombin [78]. Pradaxa (Dabigatran) and Acova (Argatroban), both univalent DTIs, are used mostly in cases of cardiovascular disease or its complications [76]. However, bleeding is the most common and serious side effect of DTIs. Thus far, DTIs are being developed, although researchers have recently focused on PAR-1 inhibition. Certain studies have reported thrombin inhibition via PAR-1-mediated platelet activation without increasing bleeding in preclinical models and small-scale clinical trials [79, 80]. Patients with acute coronary syndromes that used PAR1 antagonist, Vorapaxar (SCH530348), showed reduced risk of cardiovascular death or ischemic events in two large phase III clinical trials (Table 2). Recently, another PAR-1 antagonist, Atopaxar (E5555) used in the guinea pig model and in clinical trials, showed inhibition of PAR-1-dependent platelet aggregation and coagulation (Table 2) [81, 82]. Nevertheless, thrombin is still being studied for its relationship with local and systemic activation of coagulation and fibrinolysis pathways owing to thrombin-dependent fibrin generation and fibrin accumulation during OA pathogenesis [73]. In addition, expression of fibrin enhances inflammation and promotes cell adhesion and migration. Although the study pointed out that mediation of thrombin-induced inflammation via PAR-1 occurred in PAR-1 deficient mice, inhibition of PAR-1 had no effect on thrombin-dependent fibrin generation and coagulation [23]. However, Szaba and Smiley defined the roles for thrombin, PAR-1, and fibrinogen in a mouse peritonitis model [23]. They demonstrated that direct injection of thrombin can stimulate macrophage adhesion and peritoneal accumulation of cytokines in a fibrinogen-dependent manner* in vivo*. In further experiments with PAR-1-deficient mice, they found that thrombin stimulated vasoconstriction. Thrombin probably has pleiotropic functions, including PAR-1-mediated vasodilation, fibrin-activating macrophage adhesion, and cytokines/chemokines production during the inflammatory process [23]. In addition, Hirudin is the most potent natural inhibitor of thrombin, which possesses a specific activity to bind with the active site and fibrinogen-binding exosite 1 of thrombin and cleave fibrinogen and PAR-1 (Table 2) [83]. Some studies suggest that administration of Hirudin analogs can prevent onset and ameliorate arthritis by reducing leukocyte infiltration in a mouse glomerulonephritis model [74, 84, 85]. Taken together, the potential applications of thrombin antagonists should be explored in the treatment of OA.

### 6.2. Herbal Medicines

Many studies have demonstrated that proinflammatory cytokines and anti-inflammatory cytokines such as, IL-1, IFN-*γ*, IL-6, IL-7, IL-10, and TNF-α are expressed in OA joints [86, 87]. Curcumin (diferuloylmethane) is an anti-inflammatory, polyphenolic phytochemical. The early degenerative changes in chondrocytes were relieved by curcumin when administered as cotreatment with IL-1*β*. Additionally, collagen type II and *β*1-integrin synthesis by IL-1*β* were inhibited by curcumin [88]. Clinical studies have also shown therapeutic efficacy of curcuminoids in OA patients, where significant improvements were noted in scores for pain and physical function [89]. Therefore, curcuminoids have well-known anti-inflammatory properties and exert protective effect on chondrocytes (Table 2). *β*-Ecdysterone (Ecd), a major component of Chinese herbal medicines, is an estrogen analog, which was shown to protect chondrocytes from IL-1*β*-induced arthritis via reduction in Bax and p53 phosphorylation as well as an increase in Bcl-xL expression. Additionally, Ecd inhibited NF-*κ*B phosphorylation, I*κ*Bα degradation, and MMP-3, MMP-9, and COX-2 expression in IL-1*β*-induced arthritis. Ecd is a potent herbal medicine, used for its antiapoptotic and anti-inflammatory properties in OA (Table 2) [90]. Thrombin activity was measured by aqueous extracts from* Ceiba pentandra* (Malvaceae/Bombacoideae) and* Quassia africana* (Simaroubaceae) (*C. pentandra*). The results indicated the reduction of thrombin activity and prolonged plasma clotting time through affecting coagulation of intrinsic pathway [91]. Although there is no direct evidence that herbal medicines target thrombin in degenerative arthritis, the above results indicate both reduction of thrombin activity and extension of plasma clotting time, thus affecting the intrinsic coagulation pathway. Therefore, herbal medicines may have an adjunct nutraceutical chondroprotective application in treatment of OA and related osteoarticular disorders.

## 7. Conclusion

To conclude, we summarize that thrombin is a key factor in stimulation of fibrin deposition and proinflammatory processes in OA. Therefore, study of the thrombin signaling pathway helps us elucidate the mechanism underlying OA pathogenesis and can lead to novel therapeutic strategies in OA. Treatments that can inhibit thrombin expression and related PARs are the focus of clinical and preclinical studies that are currently underway. To date, thrombin-targeted treatment for OA has not yet reached the clinical-trial stage despite the unmet need for effective adjuvant treatments. On the other hand, a recent study showed that angiogenesis could contribute to structural damage and may act as a potential target [92]. Additionally, thrombin was shown to have increased vascular permeability, leading to tissue damage [93]. Thus, development of a specific drug targeting thrombin could inhibit angiogenesis mediators and macrophage-activated inflammatory cytokines could be explored as a therapeutic strategy for OA treatment.

## Figures and Tables

**Table 1:** Thrombin as an inflammatory-related molecule regulates signaling in OA.

Receptor	Regulation	Pathway	Function	Reference
PAR-1	CCL2	PKC/c-SRC/EGFR/MEK/ERK/AP-1	Monocytes, memory T cells, and dendritic cells activation	[47, 48]
PAR-1/-2	IL-1, TNF-*α*	p38/p42/44	MMP expression	[65]
PAR-1/-3	HO-1	PKC*δ*/c-SRC/Nrf-2	Synovial fibroblasts inflammatory	[70–72]
PAR-1/-3	MMP-13	PKC*δ*/c-SRC/EGFR/pI3K/AKT/AP-1	Cartilage destruction	[59, 60]
PAR-1	COX-2	?	IL-6, CTGF, FGF1/2, VEGF, Tnc expression	[51–54]

PAR: protease-activated receptor; CCL2: chemokine (C-C motif) ligand-2; IL: interleukin; TNF-α: tumor necrosis factor-alpha; VEGF: vascular endothelial growth factor; MMP: matrix metalloproteinase; FGF-1/-2: fibroblast growth factor-1/-2; CTGF: connective tissue growth factor; TnC: tenascin C; COX-2: cyclooxygenase-2.

**Table 2:** Thrombin as a therapeutic target in OA.

	Drug	Target	Reference
Thrombin antagonists	Vorapaxar (SCH530348)	PAR1 antagonist	[81, 82]
	Atopaxar (E5555)	PAR1 antagonist	[81, 82]
	Hirudin	Cleave fibrinogen and PAR-1	[74, 83–85]

Herbal medicines	Curcuminoids	Anti-inflammatory	[88, 89]
		Chondrocytes protection	[88]
	*β*-Ecdysterone (Ecd)	Bax and p53 reduction	[90]
		Increase Bcl-xL expression,	[90]
		Inhibited NF-*κ*B phosphorylation,	[90]
		I*κ*Bα degradation, MMP-3,	[90]
		MMP-9, and COX-2 expression	[90]
	*Ceiba pentandra* (Malvaceae/Bombacoideae)	Coagulation intrinsic pathway	[91]
		Prolong clotting time	[91]
	*Quassia africana* (Simaroubaceae/*C. pentandra*)	Coagulation intrinsic pathway	[91]
		Prolong clotting time	[91]
